# *Wolbachia* in butterflies and moths: geographic structure in infection frequency

**DOI:** 10.1186/s12983-015-0107-z

**Published:** 2015-07-16

**Authors:** Muhammad Z. Ahmed, Eli V. Araujo-Jnr, John J. Welch, Akito Y. Kawahara

**Affiliations:** Florida Museum of Natural History, University of Florida, 32611 Gainesville, FL USA; Institute of Food and Agricultural Sciences, Tropical Research and Education Center, University of Florida, 18905 SW 280th Street, 33031 Homestead, FL USA; Department of Genetics, University of Cambridge, CB2 3EH Cambridge, UK

**Keywords:** Bacteria, Butterfly, Latitudinal gradient, Moth

## Abstract

**Introduction:**

Butterflies and moths (Lepidoptera) constitute one of the most diverse insect orders, and play an important role in ecosystem function. However, little is known in terms of their bacterial communities. *Wolbachia*, perhaps the most common and widespread intracellular bacterium on Earth, can manipulate the physiology and reproduction of its hosts, and is transmitted vertically from mother to offspring, or sometimes horizontally between species. While its role in some hosts has been studied extensively, its incidence across Lepidoptera is poorly understood. A recent analysis using a beta-binomial model to infer the between-species distribution of prevalence estimated that approximately 40 % of arthropod species are infected with *Wolbachia*, but particular taxonomic groups and ecological niches seem to display substantially higher or lower incidences. In this study, we took an initial step and applied a similar, maximum likelihood approach to 300 species of Lepidoptera (7604 individuals from 660 populations) belonging to 17 families and 10 superfamilies, and sampled from 36 countries, representing all continents excluding Antarctica.

**Results:**

Approximately a quarter to a third of individuals appear to be infected with *Wolbachia*, and around 80 % of Lepidoptera species are infected at a non-negligible frequency. This incidence estimate is very high compared to arthropods in general. *Wolbachia* infection in Lepidoptera is shown to vary between families, but there is no evidence for closely related groups to show similar infection levels. True butterflies (Papilionoidea) are overrepresented in our data, however, our estimates show this group can be taken as a representative for the other major lepidopteran superfamilies. We also show substantial variation in infection level according to geography – closer locations tend to show similar infection levels. We further show that variation in geography is due to a latitudinal gradient in *Wolbachia* infection, with lower frequencies towards higher latitudes.

**Conclusions:**

Our comprehensive survey of *Wolbachia* infection in Lepidoptera suggests that infection incidence is very high, and provides evidence that climate and geography are strong predictors of infection frequency.

**Electronic supplementary material:**

The online version of this article (doi:10.1186/s12983-015-0107-z) contains supplementary material, which is available to authorized users.

## Introduction

Butterflies and moths (Lepidoptera) constitute one of the most diverse insect orders with more than 157,000 described species [1]. Lepidoptera play an important role in ecosystem function primarily as pollinators and herbivores, though some species feed on blood and other animal secretions [2–5]. The order includes many significant agricultural pests, and some species serve as models across biological disciplines [6]. Furthermore, lepidopteran larvae are hosts to another major insect radiation – the parasitic flies and wasps [7–9]. Despite the diversity of Lepidoptera and their many associations with other organisms, little is known about the bacterial community associated with the order.

*Wolbachia* (Alphaproteobacteria: Rickettsiales: Rickettsiaceae) is a genus of intracellular bacteria that infects many arthropods and nematodes [10], and is considered the most widespread endosymbiotic bacterium on Earth [10–13]. Although there is some evidence of mutualism, it is well known that *Wolbachia* can be involved in the parasitic manipulation of its host’s reproductive system [10]. *Wolbachia* is usually transmitted vertically from mother to offspring; horizontal transmission is also possible [10, 14–16]. *Wolbachia* infection frequency, both within species (prevalence) and among species (incidence), is a crucial parameter for understanding the dynamic processes behind host-endosymbiont interactions. Infection frequencies vary with transmission efficiencies, and the phenotypic effects of infection [17]. Some *Wolbachia* phenotypes increase the infection frequency in the host population whereas others decrease it [18]. Our study lays the groundwork for further biological investigations of the effects of *Wolbachia* on host Lepidoptera.

A pioneering study by Werren et al. [19] concluded that 17 % of neotropical insect species are infected with *Wolbachia*. A subsequent, expanded study by Werren and Windsor [20] concluded that at least 20 % of all insect species are infected. These studies reported sample incidence (i.e., the proportion of samples infected), but many of the samples included a small number of individuals, making low prevalence infections hard to detect. To solve this problem, Hilgenboecker et al. [11] used beta-binomial modeling to infer the between-species distribution of prevalence from the screen data. Using 20 PCR screens for *Wolbachia*, their best-fitting distribution of prevalence had peaks at very low and very high prevalence values – suggesting that many low prevalence infections were missed by previous studies. They subsequently calculated that 66 % of arthropod species were infected. Zug and Hammerstein [12] recently applied the same model to data from Duron et al. [21] and calculated a 40 % incidence in arthropods.

*Wolbachia* infection frequencies can have substantial variation both within and among particular taxonomic groups or ecological niches [19, 20, 22]. For example, Ahmed et al. [22] applied the beta-binomial approach to screening data from 172 species of fig wasps, and estimated a *Wolbachia* prevalence distribution with peaks at the two extreme values. They further estimated >80 % incidence for fig wasps, considerably higher than all previous estimates for *Wolbachia* infection frequency across arthropods [22]. Species of Lepidoptera also exhibit extreme variation in *Wolbachia* prevalence, such as the butterfly *Hypolimnas bolina* (50–100 %) [23, 24] and the moth *Plutella xylostella* (0–40 %) [25]. However, despite these studies, there is little understanding of why infection levels vary. Although much work has been conducted on *Wolbachia*-Lepidoptera interactions [19, 20, 23–29], broad patterns of *Wolbachia* prevalence and incidence in the order have not been thoroughly characterised. In this study, we synthesize prior work on *Wolbachia* in Lepidoptera and perform a new analysis of the combined data set using the beta-binomial approach. In addition, we test for variation in infection patterns between taxonomic groups and geographic regions.

## Materials and methods

We collected data from 37 studies that included *Wolbachia* screenings of Lepidoptera (Additional file 1: Table S1). To our knowledge, these studies comprise the entire literature on *Wolbachia* infection in the order. Altogether, we analyzed 300 species across Lepidoptera from 17 families and 10 superfamilies (Table 1).

**Table 1:** Summary of *Wolbachia* infection percentages in Lepidoptera

Category	No. of populations	No. of species	No. of individuals
	n (I)	n(I)	n(I)
A. Host Taxonomy (Superfamily)			
Bombycoidea	1(1.0)	1(1.0)	1(1.0)
Drepanoidea	1(1.0)	1(1.0)	1(1.0)
Gelechioidea	2(1.0)	1(1.0)	2(1.0)
Geometroidea	3(0.33)	3(0.33)	5(0.2)
Gracillarioidea	24(0.33)	20(0.35)	91(0.78)
Hepialoidea	1(1.0)	1(1.0)	4(1.0)
Lasiocampoidea	1(1.0)	1(1.0)	2(0.5)
Noctuoidea	29(0.34)	27(0.37)	129(0.42)
Papilionoidea	555(0.29)	224(0.39)	4137(0.42)
Pyraloidea	63(0.73)	19(0.78)	3013(0.19)
Tortricoidea	1(0.0)	1(0.0)	1(0.0)
Yponomeutoidea	11(0.36)	1(1.0)	306(0.05)
Total	692(0.35)	300*(0.43)	7689(0.33)
B. Host Geography (Continents)			
Africa	21(0.71)	9(0.77)	999(0.47)
Asia	326(0.28)	137(0.55)	4407(0.22)
Australia	2(0.5)	2(1.0)	78(0.03)
Europe	202(0.15)	46(0.45)	728(0.53)
North America	42(0.83)	31(0.87)	557(0.40)
Oceania	8(0.87)	1(1.0)	821(0.55)
South America	1(0.0)	1(1.0)	10(0.0)
NC	90(0.1)	89(0.10)	89(0.09)
Total	692(0.35)	316^a^(0.45)	7689(0.33)

n = total number; I = proportion infected; NC = Not calculated due to uncertainty of geographical location; ^a^21 species were infected in some populations and not in others, we considered marking a species as infected if any of its populations contained infection. Sixteen species were sampled on more than one continent (details in Additional file 1: Table S1)

### *Estimators of* Wolbachia *prevalence and incidence*

Data were analyzed using the methods described by Hilgenboecker et al. [11] and Weinert et al. [13]. This approach estimates the distribution of infection prevalences across species, whose probability density function is denoted *pdf*(*q*), and then uses this estimated distribution to calculate the mean infection prevalence

1 $$ \upmu ={\displaystyle \underset{0}{\overset{1}{\int }}pdf(q)\ q\ dq} $$

(i.e., the mean proportion of individuals infected in any given species). The distribution is also used to calculate infection incidence, i.e., the proportion of species infected above a threshold frequency of *c*. This was calculated using the formula:

2 $$ {x}_c={\displaystyle \underset{c}{\overset{1}{\int }}pdf(q)\ dq} $$

Following [11], we will mainly assume that the distribution of prevalences can be adequately described by the two-parameter beta distribution.

3 $$ pdf(q)=pdf\left(q;\upalpha, \upbeta \right)=\frac{\Gamma \left(\upalpha +\upbeta \right)}{\Gamma \left(\upalpha \right)\Gamma \left(\upbeta \right)}{q}^{\alpha -1}{\left(1,-,q\right)}^{\upbeta -1} $$

Where Γ(.) is Euler’s Gamma function and α and β are the two shape parameters. However, this distribution may be inadequate to describe the true distribution of prevalences (if, e.g., the true distribution contains a large proportion of species free from infection, and a large proportion of species with intermediate infection levels). Therefore, we follow [13] by also fitting the four-parameter doubly-inflated beta distribution:

4 $$ pdf(q)=pdf\left(q;\upalpha, \upbeta, \upgamma, \varphi \right)=\left\{\begin{array}{ccc}\hfill \upvarphi \upgamma, \hfill & \hfill q=0\hfill & \hfill \begin{array}{l}\\ {}\end{array}\hfill \\ {}\hfill \left(1-\varphi \right)\frac{\Gamma \left(\upalpha +\upbeta \right)}{\Gamma \left(\upalpha \right)\Gamma \left(\upbeta \right)}{q}^{\upalpha -1}{q}^{\upbeta -1},\hfill & \hfill q\in \left[0,1\right]\hfill & \hfill \begin{array}{l}\\ {}\end{array}\hfill \\ {}\hfill \upvarphi \left(1-\upgamma \right),\hfill & \hfill q=1\hfill & \hfill \begin{array}{l}\\ {}\end{array}\hfill \end{array}\right. $$

The parameters of these distributions, and therefore the quantities of interest (eqs. (1)–(2)) can be estimated by Maximum Likelihood, by finding the parameters that maximize the following likelihood function:

5 $$ pdf(q)=pdf\left(q;\upalpha, \upbeta, \upgamma, \varphi \right)=\left\{\begin{array}{ccc}\hfill \upvarphi \upgamma, \hfill & \hfill q=0\hfill & \hfill \begin{array}{l}\\ {}\end{array}\hfill \\ {}\hfill \left(1-\varphi \right)\frac{\Gamma \left(\upalpha +\upbeta \right)}{\Gamma \left(\upalpha \right)\Gamma \left(\upbeta \right)}{q}^{\upalpha -1}{\left(1,-,q\right)}^{\upbeta -1},\hfill & \hfill q\in \left[0,1\right]\hfill & \hfill \begin{array}{l}\\ {}\end{array}\hfill \\ {}\hfill \upvarphi \left(1-\upgamma \right),\hfill & \hfill q=1\hfill & \hfill \begin{array}{l}\\ {}\end{array}\hfill \end{array}\right. $$

where *n*_*i*_ (*k*_*i*_) is the number of individuals sampled (infected) in population *i*. This approach follows standard beta-binomial modelling when eq. (3) is used, and a full derivation and details of the numerical methods are given in [13], in which case, confidence intervals can be obtained from the curvature of the likelihood surface, as described by [30]. However, for many of the results below, we will also use the moment-based estimators of [11], in which case 95 % confidence intervals were calculated from 1000 bootstrap resamplings of the data.

### *Test for predictors of* Wolbachia *infection*

We performed Mantel tests to assess whether phylogenetic distance among lepidopteran families or geography is correlated with the absolute differences in our moment-based estimates of their mean prevalence (μ) and/or incidence (*x*_*c*_) of *Wolbachia* infection. To avoid pseudoreplication, we used randomization to calculate significance. These tests are distribution free, and so have reduced power compared to a full model-based parametric test. However, standard Brownian motion models are not appropriate for incidence and prevalence estimates, and no obvious model-based alternative exists. These tests used the “mantel.rtest” function in the “ade4” package in R version 2.13.0 [31].

To calculate geographical distance between locations, we used coordinates from the estimated midpoints of the countries where the samples were collected, and these were converted into distances using the Meeus method, which assumes an ellipsoid shape for the planet, in the R “geosphere” package [32]. To calculate evolutionary distances, we used patristic distances as found in the phylogeny of Regier et al. [33].

### Test for sample size bias

A major problem with comparative analyses is the sampling bias that arises from a focus on populations or species that are already known to contain infection [11]. To test for potential bias in our data, we used two approaches. First, we binned screens by sample size (0–10, 10–20, 20–30 etc.) and used Spearman’s rank correlation to test for an association between sample size and the estimated incidence values. Second, we used standard binomial regression to ask whether the probability an individual being infected correlated positively with the population sample size.

## Results

We assembled a data set from the literature, containing screens of 7604 lepidopteran individuals from 660 populations covering a broad taxonomic and geographic range (Table 1). Initial tests for sampling bias showed no tendency for well-sampled populations to have higher levels of infection prevalence in these data (Spearman’s rank correlation, *p* = 0.67; Slope of best-fit binomial regression = −0.00081).

### Wolbachia *incidence and prevalence in Lepidoptera*

Samples from 129 of 300 Lepidoptera species were infected with *Wolbachia*, resulting in a sample incidence of 43 % (Table 1, Additional file 2: Table S2). However, as with previous studies, these estimates are not very thorough because they ignore low prevalence infections, severely underestimating the true incidence [11].

**Fig. 1:**
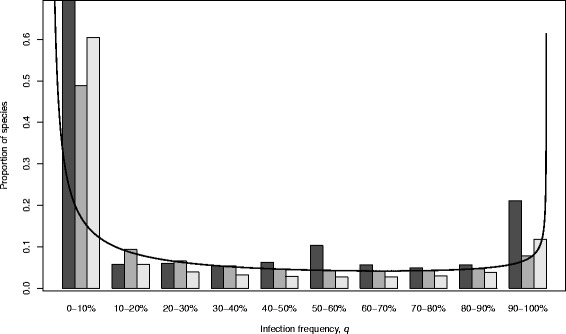
Proportion of species and infection frequencies binned in 10 % intervals. Black bars describe the observed infection frequencies within samples from each of the 312 species. For these data, the bin boundaries are treated as upper bounds (so a sample prevalence of exactly 10 % would be placed in the 0-10 % category). Dark grey bars describe the expected proportion of species infected under best-fit beta distribution as estimated by Maximum Likelihood (Table 2), and the best-fit pdf (eq. 3, with ML parameter estimates α = 0.24 and β = 0.63, scaled for visualization) is also shown for comparison. The light grey bars show the expected proportion of species under the parameter estimates of Hilgenboecker et al. [11] for their ‘B(iii)’ arthropod data set

We used maximum likelihood to fit a beta distribution to these data to estimate the between-species distribution of prevalence [11, 13]. The best-fit distribution was broadly comparable to the distribution estimated by Hilgenboecker et al. [11] for the arthropods as a whole (Fig. 1). We next used our estimated distribution to calculate the mean infection prevalence, which was μ = 28 % (CIs: 25 %–31 %), suggesting that somewhere between a quarter and a third of lepidopteran individuals are infected with *Wolbachia* on average. The distribution was next used to calculate the incidence, that is, the proportion of species that were infected above a given threshold prevalence. This analysis indicates that a large majority of lepidopteran species are infected with *Wolbachia*, with an estimated incidence of 84 % (CIs: 77 %-90 %) for a threshold of *c =* 0.001 (i.e., more than one in a thousand individuals infected; Table 2). This incidence estimate is higher than all previously published estimates across arthropods [11–13].

**Table 2:** Maximum likelihood estimates of levels of *Wolbachia* infection in Lepidoptera

Taxonomic group	Method	Mean prevalence, μ	Incidence, *x*_0.001_
Lepidoptera	Complete database; beta distribution (eq. 3)	0.28 (0.25, 0.31)	0.84 (0.77, 0.90)
	Complete database; doubly inflated distribution (eq. 4)	0.27 (0.24, 0.31)	0.77 (0.63, 1.00)
Papilionoidea	Complete database; beta distribution	0.26 (0.23, 0.29)	0.81 (0.72, 0.88)
	Complete database; doubly inflated distribution	0.25 (0.22, 0.29)	0.88 (0.65, 0.95)
	Multiple primers; beta distribution	0.32 (0.25, 0.40)	0.86 (0.72, 0.98)

Estimates are shown for the mean proportion of each population that is infected (mean prevalence), and the proportion of populations with more than 1/1000 individuals infected (incidence). Each estimate was obtained by fitting a distribution of prevalences to the screen data (see text for full details)

These estimates are open to criticism in several respects. First, the beta distribution might not be flexible enough to fit the true distribution of prevalences in nature. Indeed, the raw data (black bars in Fig. 1), suggest a trimodal distribution, which cannot be accommodated by the beta distribution (eq. (3)). To address this criticism, we followed Weinert et al. [13] and fit the more flexible doubly-inflated beta distribution (eq. (4)), which can fit trimodal distributions. This more complex distribution was favoured by model selection criterion (Akaike Information Criterion: beta 1439.79, doubly-inflated 1432.14). Nevertheless, the estimate of mean prevalence was little changed, although the estimate of incidence was reduced from 84 % to 77 % (Table 2).

Another serious criticism of our estimates is the highly biased sampling of our database, and the quality of the individual data points. As our database is dominated by true butterflies (Superfamily Papilionoidea; Table 1), we first carried out separate estimates for this Superfamily (Table 2). Again, we estimated a high incidence, which in this case was increased fitting the favoured doubly-inflated distribution (Table 2; Akaike Information Criterion: beta 1031.83, doubly-inflated 1025.271). Furthermore, this estimate was almost unchanged if we restricted our analysis to the subset of the data obtained with multiple primer sets, which are less likely to be affected by false positives due to amplification of contaminants ([34]; Table 2). Therefore, we can be relatively confident of our estimates for the true butterflies, although results reported below suggest that they may also be broadly representative of the other major lepidopteran superfamilies in our database (see below).

### Wolbachia *infection in Lepidoptera is predictable from host geography*

**Fig. 2:**
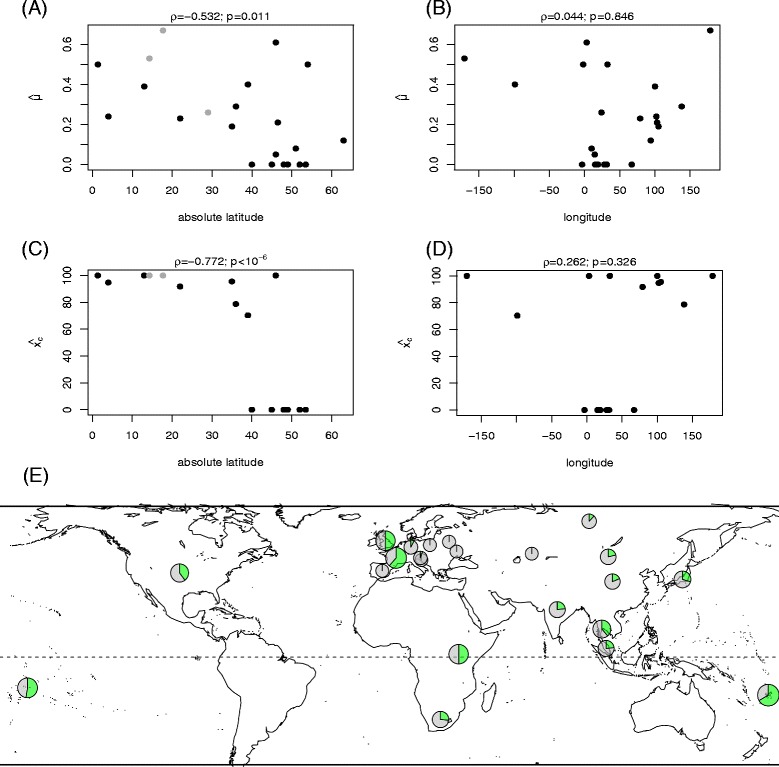
Comparison of incidence of *Wolbachia* infection and geography. **a-d** Scatter plots and Spearman’s rank correlation tests between moment-based estimators of the mean prevalence and incidence [11] and the absolute latitude and longitude of sampling locations (negative latitudes are shown as grey points). **e** The distribution of *Wolbachia* infection in Lepidoptera worldwide, based on a survey of countries with at least three screened populations. Countries/territories include: American Samoa, Belarus, China, Croatia, UK, Fiji, France, Germany, India, Japan, Kazakhstan, Malaysia, Mongolia, Poland, Russia, Slovenia, South Africa, Spain, Thailand, Uganda, Ukraine and USA. The green portion of each pie chart represents the mean prevalence (μ) in each country

Our data set of 660 lepidopteran populations includes samples from 36 countries representing four continents (Fig. 2e, Additional file 3: Figure S1). To test whether populations that are geographically proximate have similar levels of incidence or prevalence, we used a Mantel test, comparing differences in the parameter estimates for each location to the distance between those locations. Our first test included data from all 36 countries and found no significant correlation between distance and differences in mean prevalence (*n =*36; μ: *r* = 0.002, *P =* 0.42). However, several of these countries were sparsely sampled, meaning that prevalence estimates are likely imprecise. Our second test excluded these poorly sampled countries (i.e., those with fewer than three populations screened) and indicated a highly significant relationship (*n =*23; μ: *r* = 0.31, *P* = 0.006). Our test for incidence also excluded these poorly sampled countries, as well as all countries where the moment-based estimators of Hilgenboecker et al. [11] yielded negative (and therefore nonsensical) estimates of the variance in infection prevalence. We found that geographic proximity was a highly significant predictor of infection incidence (*x*_*0.001*_: *r* = 0.25, *P* = 0.009; *x*_*0.0001*_: *r* = 0.23, *P* = 0.01). To explain this result, we plotted estimated prevalence and incidence against absolute latitude and longitude (Fig. 2a-d). Rank correlation tests showed a highly significant relationship for both incidence and prevalence with absolute latitude, but no clear pattern with longitude. Therefore, we infer that *Wolbachia* infection varies predictably with latitude, with populations closer to the equator having higher prevalence and incidence.

#### *No phylogenetic signal for* Wolbachia *infection in Lepidoptera*

**Fig. 3:**
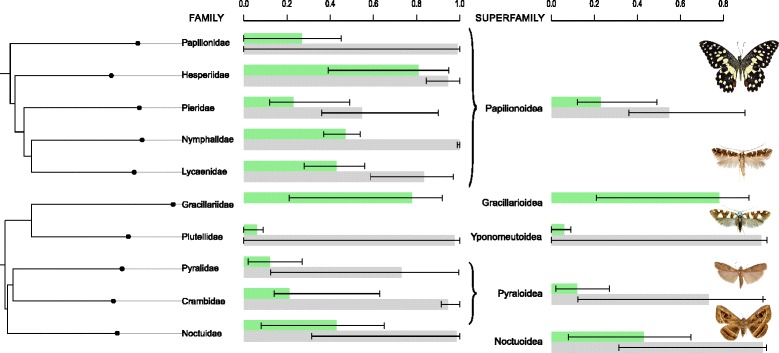
Distribution of *Wolbachia* mapped on the Lepidoptera phylogeny of Regier et al. [33] Moment-based estimators of prevalence (μ), and incidence (x_c_), are shown in green and grey respectively. For Gracillarioidea and Gracillariidae, the moment-based estimation method rendered nonsensical negative parameter estimates, and so these are not shown

We obtained estimates for incidence and prevalence for each of the sampled superfamilies and families of Lepidoptera to test the effect of phylogeny on infection rate. Confidence intervals on these estimates (Fig. 3) suggested that there are significant differences in prevalence between taxonomic groups, and significant difference in incidence between families, but not superfamilies.

To determine whether these estimates showed phylogenetic signal (i.e., whether there was a tendency for closely-related lepidopteran groups to show similar levels of incidence and prevalence), we used a Mantel test on the family-level data, comparing differences in levels of *Wolbachia* infection to the phylogenetic distance between the families. While correlations were positive, we found no significant relationship in either prevalence or incidence case (*n* =10; μ: *r* = 0.12, *P* = 0.20; *n =* 9*; x*_*0.001*_: *r* = 0.054, *P* = 0.43; *x*_*0.0001*_: *r* = 0.067, *P* = 0.41) (Fig. 3).

## Discussion

Lepidoptera are among the most diverse orders of insects, with more than 157,000 described species [1]. Lepidoptera are also among the most widely distributed, inhabiting all terrestrial biomes except Antarctica. Their larvae are predominantly herbivores, and their adults play a key role in ecological systems as pollinators [35]. Some species rank among the most destructive agricultural pests, causing significant damage to crops, stored products and natural forests [36]. There is evidence that *Wolbachia* in Lepidoptera have both parasitic and mutualistic relationships [17, 23, 37–40]. A well-known effect of *Wolbachia* in Lepidoptera is reproductive manipulation, including feminization, androcide, and cytoplasmic incompatibility [23, 37, 38]. One species of *Wolbachia* enhances the susceptibility of its lepidopteran host to baculovirus, rendering it a potential biological control agent against the agricultural pest *Spodoptera exempta* [40]. In the current study, we demonstrate that *Wolbachia* infects a high proportion of Lepidoptera individuals and species, reflecting the significance of *Wolbachia*’s role in moth and butterfly biology [10].

Our global survey of published screenings of over 300 lepidopteran species found that 43 % of samples were infected with *Wolbachia* (Table 1), a substantially higher estimate than most previous localized reports: 16.2 % (*n* = 43 species [19]) from Panama, 35.2 % (*n* = 34 [26]) from the UK, 14.3 % (*n* = 21 [20]) from the US, 17 % (*n* = 24 [27]) from Uganda, 45 % (*n* = 49 [28]) from Japan, 43 % (*n* = 7 [21]) from Western Europe, and 52 % from India (*n* = 56 [29]).

We also expanded on previous studies by using a model-based analysis to estimate infection frequency across the order. Using a beta-binomial approach to estimate the distribution of prevalence across species [11], we estimated that the vast majority of Lepidoptera are infected with *Wolbachia* (around 80 %), a much greater frequency than has been estimated across arthropods as a whole [11, 12]. However, the mean prevalence in Lepidoptera reported here (27 % in the preferred model) does not significantly differ from the estimated prevalence in arthropods: the value reported by Hilgenboecker et al. [11] (25.3 %) falls within our confidence interval boundaries (24-31 %). The very high incidence that we estimate may reflect the opportunities for substantial horizontal transfer of *Wolbachia* in Lepidoptera. It must be pointed out that our results come from a data base consisting largely of true butterflies (Papilionoidea, comprising 83 % of the 660 Lepidoptera populations used). However, the results suggest that true butterflies might not be unrepresentative of the other major lepidopteran superfamilies, from which they do not differ significantly in our estimates (Fig. 3). This homogeneity in high frequency infection across groups makes sense in light of the fact that the majority of lepidopteran larvae feed on plant tissue and that adults obtain nectar from flowers or tree sap [41], if these food sources are possible ways to mediate cross-infection in different populations and species [42].

In arthropods, bacterial infection frequencies can be influenced by abiotic factors, such as geographical location and climate condition [43–46], or by biotic factors such as host genetic variation and competition with other endosymbionts on the same host [47, 48]. We found no correlation between *Wolbachia* infection frequency and phylogenetic relatedness of lepidopteran host groups. However, there was a significant correlation between infection frequency and host geography. *Wolbachia* infection of lepidopteran species has been known to differ between geographical regions [20], though not substantially, and other arthropod groups have shown no evidence of geographical variation [20, 22]. Our study revealed that *Wolbachia* infection frequencies in Lepidoptera are higher at lower absolute latitudes, suggesting that infection is greater in warmer climates (Fig. 2). This result is in agreement with the findings of Toju and Fukatsu [45], who observed high *Wolbachia* infection frequencies in weevils that were found in climates with higher temperature. However, Liu et al. [44] and Morag et al. [46] found that the higher infection frequencies occur in regions with moderate climatic conditions, as opposed to geographic regions with extreme climates. Furthermore, Sumi et al. [49] found no seasonal effect on *Wolbachia* infection in the butterfly *Pseudozizeeria maha* (Lycaenidae). Our results seem difficult to reconcile with experimental demonstrations that increasing temperatures reduce *Wolbachia* infection frequencies [50–53]. However, latitudinal gradients are also correlated with additional factors, such as species densities and their interactions [54], so there are other plausible explanations for the correlation between *Wolbachia* infection and absolute latitude, such as climatic factors. This study serves as a foundation for future research that can provide more insight into the factors that impact *Wolbachia* and its interactions with Lepidoptera.

## Conclusion

We collated data from what we believe are all published sources of *Wolbachia* infection in Lepidoptera. We estimate that around 80 % of species and from a quarter to a third of individuals are infected at non-negligible frequency. We found no evidence that closely related taxonomic groups show similar infection levels, but we did find geographical variation, with closer locations tending to show similar infection levels. We also show that latitudinal gradient appears to be an important factor in infection level, with lower frequencies towards higher latitudes. Our study is the first to show such a latitudinal gradient in *Wolbachia* infection at such a broad taxonomic and geographic scale, and suggests that symbiont infection might be predictable from ecological variables.

## Additional files

Additional file 1: Table S1. Survey of *Wolbachia* infection in Lepidoptera. The details of host taxonomy, its geography and its infection status along with references. Additional file 2: Table S2.
*Wolbachia* infection frequency estimates and beta-binomial parameter estimates for geographic and taxonomic groups of Lepidoptera. Additional file 3: Figure S1. Distribution of *Wolbachia* mean prevalence (a and b) and incidence (c and d) across global regions and countries. Missing incidence values indicate groups whose data rendered nonsensical parameter estimates.
