# Supplementary figures and images for: Wolbachia in butterflies and moths: geographic structure in infection frequency
Source: Front Zool. 2015 Jul 16;12:16. doi: 10.1186/s12983-015-0107-z (PMC4502936; doi:10.1186/s12983-015-0107-z)

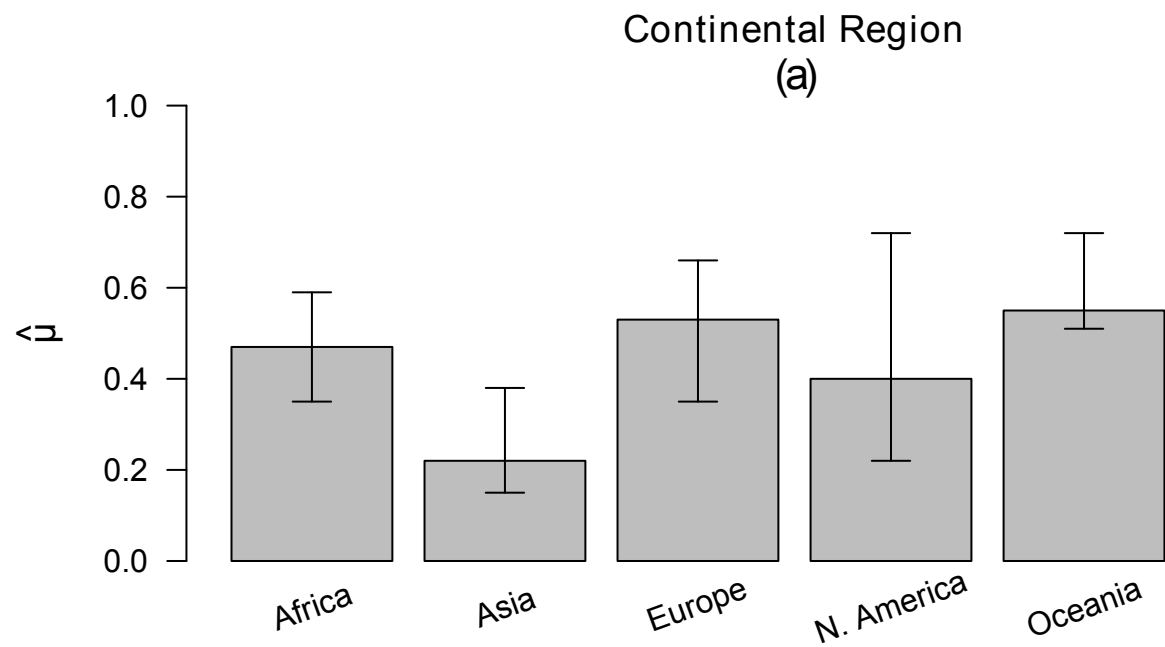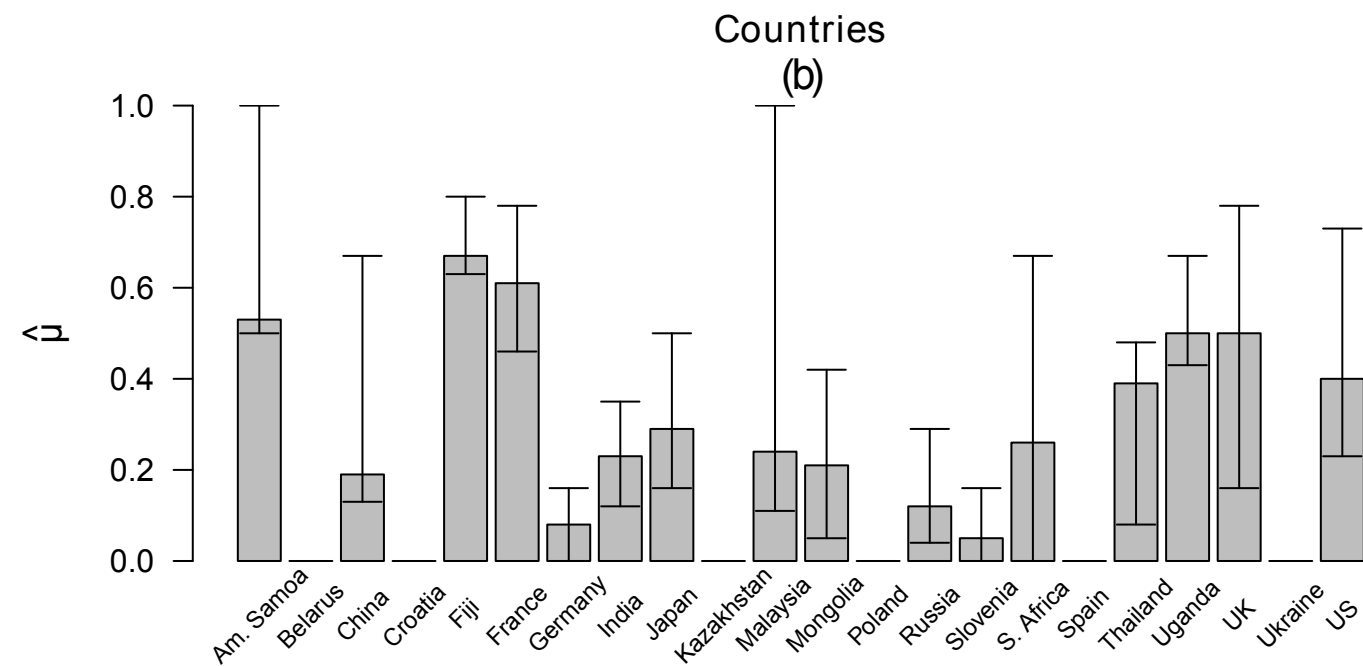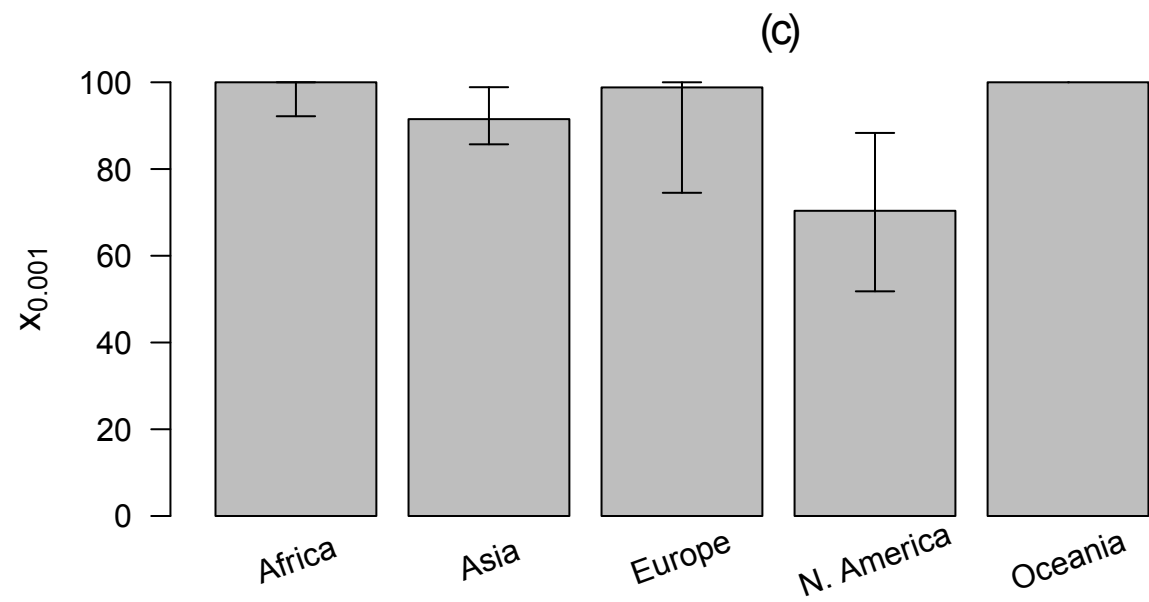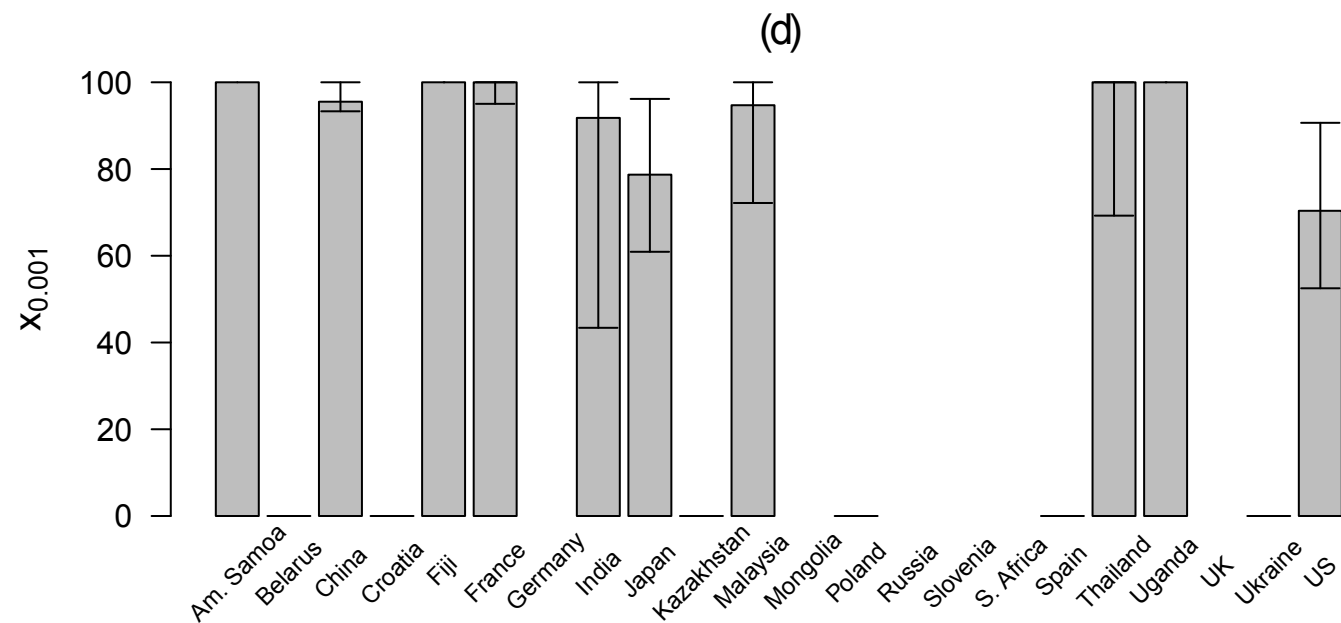

Supplement: Additional file 3: Figure S1. — Distribution of Wolbachia mean prevalence (a and b) and incidence (c and d) across global regions and countries. Missing incidence values indicate groups whose data rendered nonsensical parameter estimates. [file 12983_2015_107_MOESM3_ESM.pdf]
